# Predicting Response to Vedolizumab in Inflammatory Bowel Disease

**DOI:** 10.3389/fmed.2020.00076

**Published:** 2020-04-02

**Authors:** Joseph Meserve, Parambir Dulai

**Affiliations:** Department of Gastroenterology, University of California, San Diego, San Diego, CA, United States

**Keywords:** vedolizumab, biologic, response, prediction model, IBD

## Abstract

Vedolizumab is known to be safe, well-tolerated, and effective. However, as personalization becomes an increasingly important aspect of IBD care and in lieu of guidelines to inform clinicians on positioning of biologics, there is a need to reliably predict response to inform patient preferences and shared decision-making. Recent data from clinical trials and real-world evidence have elucidated predictors of clinical and endoscopic response while providing the framework to establish predictive models. Current models are able to predict that those patients with less severe disease, without prior biologic exposure and who demonstrate early response to VDZ have the highest rates of durable clinical and endoscopic response and remission. When incorporating these models into clinical practice, clinicians will be able to identify those patients who are likely to respond before drug initiation as well as early non-responders and response latency after initiation of vedolizumab. In a shift toward personalization of medicine in IBD, the ability of predictive models for vedolizumab to aid pre-biologic and early management will inform both clinician and patient. Ideally this will provide both a personalized and more cost-effective approach, though further studies in cost-analysis in this framework are needed. Though current models are comprehensive of existing data, future research on microbial and translational biomarkers will be additive and necessary to provide full personalization of treatment.

## Key Concepts

- Vedolizumab is safe, well-tolerated, and effective.
- UC and CD patients with less severe disease, without prior biologic exposure, and who demonstrate early response to VDZ are most likely to respond to therapy.
- The CDST from Dulai et al. can be used before initiation of VDZ to determine those most likely to respond and those who may be more likely to benefit from early consideration of dose escalation or alternative therapy.
- The CDST from Dulai et al. was able to predict drug exposure, rapidity of onset, and clinical outcomes including clinical and steroid-free remission.

## Introduction

Vedolizumab (VDZ) is a humanized monoclonal anti-integrin biologic approved for moderate to severe Crohn's Disease (CD) and Ulcerative Colitis (UC). Vedolizumab selectively inhibits leukocyte extravasation into the gut, and few other less clinically relevant tissues, via disruption of alpha4beta7 integrin on leukocytes and adhesion molecules on the vascular endothelium. Phase 3 clinical trials confirmed the efficacy of VDZ in CD and UC and observational cohorts have confirmed its real-world effectiveness and safety. Despite the favorable safety profile and effectiveness of VDZ there are no guidelines to aid clinicians with its positioning among biologics. The ushering in of the biologic era brought with it the luxury of greater choice. With multiple options available for therapy in moderate to severe Inflammatory Bowel Diseases (IBD), many of which appear to be equivalent in effectiveness and safety, there has been a necessary push to improve shared-decision making around treatment choices. Hierarchical preferences of providers and patients could bring traditionally second-line therapies to the forefront. With personalization of therapy to these preferences and without formal guidelines or robust comparative clinical trials, it will be increasingly important for clinicians to critically evaluate existing data for many treatment-related factors, including predicting response. In this article we will review current literature from clinical trials, their *post-hoc* analyses, and real-world data that elucidate predictors of primary response to VDZ in CD and UC.

## Predictors of Clinical Response and Remission

### Baseline Disease Activity

Subgroup analyses of the GEMINI 1 and 2 trials evaluated demographic and baseline characteristics associated with response and/or remission at 6 and 52 weeks. Less severe clinical disease scores, CDAI score ≤ 330 and Mayo score <9, were associated with higher likelihood of remission compared to placebo at 6 and 52 weeks in CD and UC (1, 2). Real-world observational cohorts have supported this finding. The US VICTORY consortium found that those patients with baseline clinically severe CD or active perianal disease were less likely to obtain clinical remission (3). The French GETAID cohort found that patients with more severe baseline UC or CD were less likely to achieve clinical remission at 14 and 54 weeks (4, 5). An Israeli cohort reported that mild clinical disease activity was associated with increased clinical remission in CD at 14 weeks, with no predictors in UC (6). A German cohort of 97 CD patients found that a low Harvey-Bradshaw Index (HBI) score and no hospitalizations in the preceding year predicted clinical remission at 14 weeks (7). In the largest cohort assessed, Chaparro et al. found that higher baseline HBI in CD to be a negative predictor and mild disease in UC to be a positive predictor of clinical remission at 14 weeks (8) (Table 2).

### TNF Antagonist Exposure

It's known that efficacy of TNF antagonists is lower with a second agent after loss of response to a first, and it could be expected that this would be seen with other biologics following TNF antagonist therapy (9, 10). In a pooled *post-hoc* analysis of GEMINI 2 and 3, TNF antagonist naïve patients who had responded to VDZ at 6 weeks were more likely to achieve or maintain remission at week 52 as compared to TNF antagonist failure patients (11) (Table 1). Sands et al. found that patients with CD who had failed TNF antagonist therapy were more likely to be in clinical remission at 10 weeks but not 6 weeks as compared to placebo (26.6 vs. 12.1% [*p* = 0.001] and 15.2% vs. 12.1% [*p* = 0.433]) (14). The VICTORY consortium observed that prior TNF antagonist exposure was associated with lower rates of remission and mucosal healing in CD and decreased rates of response and remission in UC, and this observation remained irrespective of the statistical approach applied to the data (15). Similarly, results from Stallmach et al. demonstrated that TNF antagonist exposed UC patients were less likely to achieve clinical remission (16). An Israeli cohort in contrast found that prior TNF antagonist exposure had no effect on outcomes of UC or CD at 52 weeks, though limited by low numbers of TNF-naïve patients (8%) (17) (Table 2).

**Table 1:** *Post-hoc* analysis of GEMINI trials.

**References**	**Cohort**	**Outcomes**
Feagan et al. (12)	*Post-hoc* analysis of GEMINI 1	**Week 6 Clinical Remission (TNF-naïve):** VDZ 23.1% vs. Placebo 6.6% (RR = 3.2; 95% CI 1.3–7.9) **Week 6 Clinical Remission (TNF-failure):** VDZ 9.8% vs. Placebo 3.2% (RR = 3.2; 95% CI 0.7–14.5) **Week 6 Mucosal Healing (All Patients):** VDZ 40.9% vs. 24.8% (RR = 1.6; 95% CI 1.2–2.3) **Week 52 Clinical Remission (TNF-naïve):** VDZ 53.1% vs. Placebo 26.2% (RR = 2; 95% CI 1.3–3) **Week 52 Clinical Remission (TNF-failure):** VDZ 36.1% vs. Placebo 5.3% (RR = 6.6; 95% CI 1.7–26.5) **Week 52 Mucosal Healing (All Patients):** VDZ 53.8% vs. Placebo 19.8% (RR = 2.7; 95% CI 1.9–4)
Sands et al. (11)	*Post-hoc* analysis of GEMINI 2 and 3	**Week 6 Clinical Remission (TNF-naïve):** VDZ 12.6% difference from placebo (95% CI 3.7–21.4) **Week 6 Clinical Remission (TNF-failure):** VDZ 4.1% difference from placebo (95% CI −1.6–9.8) **Week 52 Clinical Remission (TNF-naïve):** VDZ 22.1% difference from placebo (95% CI 8.9–35.4) **Week 52 Clinical Remission (TNF-failure):** VDZ 14.9% difference from placebo (95% CI 4.7–25)
Sands et al. (13)	*Post-hoc* analysis of GEMINI 2 and 3	**GEMINI 2 Week 6 Remission:** VDZ+CS 19.0% vs. Placebo+CS 4.6% (14.4% difference; 95% CI −1.3–29.6) VDZ 10.9% vs. Placebo 8.6% (without CS) (2.3% difference; 95% CI −6–10.6)**GEMINI 3 Week 6 Remission:** VDZ+CS 198% vs. Placebo+CS 10.2% (9.6% difference; 95% CI 0.3–19) VDZ 18.6% vs. Placebo 14.4% (without CS) (4.1% difference; 95% CI −6.3–14.6)

*CD, Crohn's Disease; UC, Ulcerative Colitis; RR, Relative Risk; CI, Confidence Interval; CS, Corticosteroid; IS, Immunosuppression; TNF, Tumor Necrosis Factor; SES-CD, Simple Endoscopic Score for Crohn's Disease; CRP, C-reactive Protein; CDAI, Crohn's Disease Activity Index*.

**Table 2:** Predictors of clinical response to VDZ from real-world cohorts.

**References**	**Cohort**	**Outcomes**	**Positive predictors of response**	**Negative predictors of response**
Amiot et al. (4)	272 patients (161 CD) with prior conventional or TNF antagonist therapy who completed induction. A multicenter French cohort	Steroid-free clinical remission at 54 weeks (HBI ≤4 or partial Mayo score <3 with a combined stool frequency and rectal bleeding subscore of ≤1)	CD: Week 6 response (OR = 7.41; 95% CI 2.85–19.23) UC: Week 6 response (OR = 7.51; CI: 95% 3.00–18.88)	CD: Corticosteroids at induction (OR = 0.37; 95% CI 0.16–0.88). HBI score > 10 at induction (OR = 0.15; 95% CI 0.06–0.37)UC: WBC > 9000 × 109/L (OR = 0.36; 95% CI 0.14–0.92). Mayo score > 9 at induction (OR = 0.37; 95% CI 0.15–0.92)
Amiot et al. (5)	294 patients (173 CD) with prior conventional or TNF antagonist therapy. A multicenter French cohort	Steroid-free clinical remission at 14 weeks (HBI ≤4 or partial Mayo score <3 with a combined stool frequency and rectal bleeding subscore of ≤1)	CD: Week 6 response (OR = 11.2; 95% CI 4.3–28.8; *p* = < 0.001) UC: Week 6 response (OR = 5.3; 95% CI 2.2–13.1; *p* = < 0.001)	CD: Corticosteroid use at induction (OR = 0.35; 95% CI 0.16–0.77; *p* = 0.009). HBI score > 10 at induction (OR = 0.11; 95% CI 0.05–0.27; *p* = < 0.001)UC: CRP > 20 mg/L at induction (OR = 0.30; 95% CI 0.11–0.80; *p* = 0.02). Mayo score > 9 at induction (OR = 0.21; 95% CI 0.08–0.57; *p* = 0.002)
Baumgart et al. (7)	212 patients (97 CD) eligible for VDZ. Single site, prospective, German cohort	Clinical remission at 14 weeks (HBI ≤4 or partial Mayo score ≤1 plus a bleeding subscore of 0)	CD: Low HBI score (*p* = 0.02). No hospitalization in prior year (*p* = 0.01) UC: No predictors	
Chaparro et al. (8)	521 patients (259 CD) with ≥1 induction VDZ dose. Multicenter Spanish cohort	Clinical remission at 14 weeks (partial Mayo score <2 or HBI score <5)	UC: Mild vs. severe disease (OR = 6.6; 95% CI 3–14.7)	CD: Higher baseline HBI (OR = 0.6; 95% CI 0.5–0.7)UC: Higher baseline CRP (OR = 0.8; 95% CI 0.8–0.9)
Dulai et al. (3)	212 CD patients eligible for VDZ from a multicenter US cohort	Clinical remission (complete resolution of all CD-related symptoms)		Prior TNF-antagonist exposure (HR = 0.40; 95% CI 0.20–0.81)Active or historical smoking (HR = 0.47; 95% CI 0.25–0.89)Active perianal disease (HR = 0.49; 95% CI 0.27–0.88)Severe disease activity (HR 0.54; 95% CI: 0.31–0.95)
Dulai et al. (18)	180 UC patients eligible for VDZ from a multicenter US cohort	Clinical remission (complete resolution of all UC-related symptoms) and response (clinically significant response defined as >50% reduction in symptom activity by PGA)		Achieve response with prior TNF-antagonist exposure (HR, 0.58; 95% CI, 0.39–0.86)Achieve remission with prior TNF-antagonist exposure (HR, 0.55; 95% CI, 0.35–0.88)
Kopylov et al. (6)	204 patients (130 CD) treated with VDZ with at least 14 weeks of follow-up from a multicenter Israeli cohort	Clinical remission at 14 weeks (HBI <5 and a partial Mayo score <2 or SCCAI <4)	CD: Mild clinical activity at induction(*p* = 0.001) UC: no predictors	
Kopylov et al. (17)	193 patients (133 CD) who completed 52 weeks of VDZ treatment with follow-up from a multicenter, retrospective, Israeli cohort	Clinical remission at 52 weeks (HBI ≤4, CDAI <150; SCCAI <2, partial Mayo score ≤2)	CD: Clinical response at 14 weeks (OR = 3.5; 95% CI 1.4–8.6) UC: Clinical response at 14 weeks (OR = 7.3; 95% CI 1.8–29.1)	
Lenti et al. (19)	203 patients (135 CD) treated with VDZ from a multicenter UK retrospective cohort	Clinical response and remission at 14 and 52 weeks (partial vs. complete/significant symptom relief by PGA)	No predictors	
Shelton et al. (23)	172 patients (107 CD) receiving ≥3 VDZ infusions at 2 US academic centers	Clinical response and remission at 14 weeks	Baseline CRP >8.0 mg/L (OR = 0.33; 95% CI 0.15–0.95. *p* = 0.04)	
Stallmach et al. (16)	127 patients (67 CD) eligible for VDZ from a single site, prospective German cohort	Clinical remission at 54 weeks (HBI ≤4 or a partial Mayo score ≤1 with a bleeding subscore of 0)	CD: Response or remission at week 14 (*p* = < 0.001). Lower CRP at week 14 as compared to baseline (*p* = 0.01) UC: Remission at week 14 (*p* = < 0.0001). No prior TNF antagonist treatment (OR = 5.3; 95% CI 1.3–21.4). Less than 25% use of steroids within prior 6 months (OR = 5.4; 95% CI 1.3–22.1). Lower CRP at week 14 as compared to baseline (*p* = 0.003). Lower fecal calprotectin at week 14 (*p* = 0.002)	

*CD, Crohn's Disease; UC, Ulcerative Colitis; OR, Odds Ratio; HR, Hazard Ratio; CI, Confidence Interval; HBI, Harvey-Bradshaw Index; SCCAI, Simple Clinical Colitis Activity Index; CRP, C-reactive Protein; CDAI, Crohn's Disease Activity Index; PGA, Physician's Global Assessment*.

### Concomitant Immunosuppressive Therapy

The GEMINI trials were not powered to assess combination therapy, however, sub-group analyses did not observe a difference between VDZ monotherapy and combination therapy on rates of response or remission (1, 2). Real-world cohorts observed that steroid use was associated with lower rates of response in CD (5, 6) and UC (4, 16), possibly a confounding due to indication as steroids are more often used in patients with more severe disease, but immunomodulator addition after induction was associated with increased response and remission in CD (16). These data did not bear out in remaining real-world cohorts. For example, no differences were noted with any concomitant therapy in Israeli cohorts or the VICTORY or Cross Penine cohorts (3, 17–19). Regardless of these results, it is important to remember that the appeal of the relative safety for VDZ is decreased with combination therapy with corticosteroids and/or immunomodulators (20), and there also does not appear to be the same risk of immunogenicity or benefit of increased trough levels with concomitant immunomodulators for VDZ (21, 22) (Table 2).

### Biomarkers

GEMINI 1 and 2 demonstrated that elevated inflammatory markers were associated with lower rates of clinical response and remission (1, 2) and data from real-world cohorts support this finding. A 172 cohort of UC and CD patients from a pair of Boston academic centers observed that rates of remission were lower with elevated CRP (23). The French GETAID cohort shared this finding for patients with UC (5). Stallmach et al. found that an early (week 14) reduction in CRP or fecal calprotectin was associated with higher rates of remission at 54 weeks (16).

However, biomarkers assessed in current trials and real-world cohorts are nonspecific and related to overall disease activity. Battat et al. reviewed novel biomarkers which were postulated to be associated with VDZ response in UC due to their potential relationship to the α4β7 and adhesion molecule interaction that is inhibited by VDZ (24). At induction, lower soluble TNF was associated with achieving remission. During maintenance, lower soluble VCAM-1 and higher soluble α4β7 were associated with achieving remission. These results are promising and suggest that novel biomarkers could be incorporated into future studies and prediction models to improve VDZ-specific response prediction (Table 2).

### Microbiome

The gut microbiome is known to be associated with mucosal inflammation in IBD. Ananthakrishnan et al. recruited a prospective cohort of 42 CD and 43 UC patients receiving VDZ and assessed microbial composition related to disease activity (25). Changes in microbiome diversity were associated with clinical remission in those with CD but not UC. Assessment of biochemical pathways revealed a significant increase in week 14 remission in patients with CD who had baseline enrichment of BCAA pathways, suggesting a functional component in addition to taxonomic differences as baseline predictors. Of note, the microbial changes of those who achieved remission at week 14 persisted at 1 year, suggesting an early marker rather than a baseline predictor of response. While this study suggests multiple microbial markers of baseline and early predictors of response to VDZ (ie taxonomic differences, diversity, and function) it lacks applicability as microbiome sequencing has not reached clinical point of care. It is also limited by its small, single-center cohort with limited follow-up and assessment of diet and would require further validation; but nonetheless an interesting pilot study to complement the data regarding TNF effect on microbiota and worth further investigation.

## Predictors of Endoscopic Response or Remission

Endoscopic response is an important part of disease assessment and is becoming a larger part of the treatment target in IBD. The recent VERSIFY phase 3b clinical trial (26) assessed endoscopic response to VDZ in CD and found that endoscopic remission rates (SES-CD score ≤ 4) were greater in patients naïve to TNF antagonists, those with moderate compared to severe baseline endoscopic disease, and shorter disease duration (26). Endoscopic remission rates at week 26 and 52 were higher in TNF-antagonist naïve (9.6 and 25%) vs. TNF-antagonist exposure (5.5 and 8.3%), higher in moderate disease (SES-CD 7-15) (17 and 20.7%) vs. severe disease (SES-CD > 15) (6.7 and 14.8%), and higher in shorter disease duration (<1 year) (37.5 and 100%) vs. longer disease duration (≥7 years) (7.1 and 11.5%).

*Post-hoc* analysis of the GEMINI 1 trial found that mucosal healing rates (Mayo endoscopic subscore of ≤1) were higher among VDZ treated patients with UC at 6 weeks (RR = 1.6; 95% CI 1.2–2.3) and 52 weeks (RR = 2.7; 95% CI 1.9–4) as compared to placebo (12).

The VICTORY cohort evaluated endoscopic response to VDZ in UC and found that 17% of patients achieved endoscopic remission (Mayo endoscopic sub-score 0) at 12 months. Prior TNF-antagonist was associated with reduced probability of achieving endoscopic response (HR 0.51, 95% CI 0.29–0.88) (18).

In a Canadian real-world cohort evaluating endoscopic and radiologic remission, VDZ patients with CD were less likely to obtain objective remission at 6 months (adjusted OR 0.30; 95% CI: 0.11–0.79, *p* = 0.02) and 12 months (adjusted OR 0.27; 95% CI: 0.09–0.78, *p* = 0.02) compared to UC (27). There were no differences in rates of remission due to disease severity, previous biologic failure, and pretreatment of CRP. Of note, this study did not separate endoscopic and radiographic remission.

## Predictors of Adverse Events

Colombel et al. provided an integrated VDZ clinical trial analysis from the GEMINI trials and their follow-up long-term safety data (>4000 PYs) (28). They found VDZ to be well-tolerated with an acceptable safety profile. Overall, patients with UC and CD exposed to VDZ had less adverse events (AE) than placebo when adjusted for exposure (247.8/100 vs. 419.4/100 PYs). This included infectious AEs, with overall incidence in VDZ-exposed being lower than placebo (63.5/100 vs. 82.9/100 PYs). Due to the gut-selective mechanism of action of VDZ there may be concern that these patients are at higher risk for enteric infections. However, the rates of enteric infections were very low (≤ 0.8/100 PYs), excluding gastroenteritis. Predictors of serious infection in total cohort of UC and CD were younger age, opioid use, and corticosteroid use. When separated by type of IBD, prior TNF-antagonist failure was found to be a predictor of serious infection in the UC cohort but not younger age or concomitant steroid use.

In our analysis of real-world data from the VICTORY cohort, we also found VDZ to be well-tolerated with a similar safety profile to the GEMINI trials (20). Predictors of infection included active smoker status and number of concomitant immunosuppressive agents. VDZ monotherapy and VDZ plus immunomodulator had comparable rates of AEs (5.9/100 vs. 5.8/100 PYE), but the addition of corticosteroids to either resulted in increased risk of infection in an incremental fashion (VDZ+CS 9.5/100 PYE vs. VDZ+IM+CS 12/100 PYE). This is important to note and discuss with patients as the gut-selective mechanism of VDZ is thought to convey this favorable safety profile which cannot be relied on with the addition of other immunosuppressants.

## Prediction Modeling

There are many potential predictors of response that have been identified from clinical trial and real-world data (see Tables 1, 2 for summary), however, translating these findings into clinical practice can be challenging. The ability to cluster these data into a tool that can inform patients and clinicians about potential response early in treatment course, or ideally before starting, would allow for greater personalization within IBD therapy. Waljee et al. and Dulai et al. have both developed prediction models of response from *post-hoc* analyses of the GEMINI trials to address this need. Although both used a similar dataset for model derivation, differences exist between them which are important to highlight.

First, Waljee et al. utilized a machine-learning approach that incorporated baseline patient characteristics and labs in combination with changes in lab values during induction (29, 30). Our group in contrast used regression methodology with a primary focus on baseline patient characteristics and labs (31, 32). This distinction is important because the machine-learning model therefore requires a trial of induction therapy prior to determining if a patient is likely to respond to VDZ whereas baseline regression models can help classify patients before treatment initiation thereby avoiding the need to prove a lack of response or sub-optimal response after induction. Second, both groups used corticosteroid-free clinical remission and endoscopic remission as dependent outcomes for CD and UC, but our model also incorporated predictors of clinical remission and durable remission for CD into the assessment. Third, both groups transformed these models into clinical decision support tools (CDST) with Waljee et al. creating a simplified equation using variable importance plots and our group creating a point scoring system based CDST. Fourth, although both models demonstrated modest accuracy and performance within the GEMINI cohort, only the regression models underwent external validation in routine practice cohorts of patients treated with VDZ. Finally, the regression-based prediction models and CDSTs have now been shown to be able to predict not only clinical and endoscopic effectiveness, but also rapidity of treatment response, measured drug exposure, and biomarker response; thereby providing a more comprehensive prediction of key components to patient outcomes and opportunities for treatment optimization (Table 3).

Table 3Prediction models.

**Table:** 

	**Regression Models (Dulai)**		
	**CD**		**UC**
**Derivation-GEMINI Cohorts**			
Performance	Week 26 CREM AUROC 0.69 Week 26 CSF-REM AUROC 0.69 Week 52 CREM AUROC 0.68		Week 52 CSF-REM AUROC 0.69
**Validation-VICTORY Cohorts**			
Primary Outcome	Clinical and Endoscopic Remission at Week 26	Sensitivity/Specificity (95% CI) of CDST at Week 26	Sensitivity/Specificity (95% CI) of CDST at Week 26
Performance	Week 26 CREM AUROC 0.67 Week 26 CSF-REM AUROC 0.66 Week 26 Mucosal Healing AUROC 0.72 Week 26 Deep remission AUROC 0.73 Week 26 CF-REM with MH AUROC 0.75	**13 points:** CREM Sensitivity: 92% CREM Specificity: 25% CSF-REM Sensitivity: 94% CSF-REM Specificity: 30% MH Sensitivity: 98% MH Specificity: 30% CSF-DR Sensitivity: 100% CSF-DR Specificity: 31% **19 points:** CREM Sensitivity: 33% CREM Specificity: 80% CSF-REM Sensitivity: 37% CSF-REM Specificity: 77% MH Sensitivity: 40% MH Specificity: 80% CSF-DR Sensitivity: 46% (19–75%); CSF-DR Specificity: 78% (69–85%)	**26 points:** CSF-REM Sensitivity: 93% CSF-REM Specificity: 15% **32 points:** CSF-REM Sensitivity: 51% CSF-REM Specificity: 68%
POC Transformation	Absence of prior TNF antagonist exposure (+3 points) Absence of prior bowel surgery (+2 points) Absence of prior fistulizing disease (+2 points) Baseline level of albumin (+0.4 points per g/L) Baseline concentration of C-reactive protein (reduction of 0.5 points for values between 3.0 and 10.0 mg/L and 3.0 points for values >10.0 mg/L)		Absence of prior TNF antagonist exposure (+3 points) Disease duration ≥2 years (+3 points) Baseline endoscopic activity (moderate vs. severe) (+2 points) Baseline albumin concentration (+0.65 points per g/L)

**Table:** 

**Secondary Outcomes from Dulai Prediction Models**						
			**Low probability**	**Intermediate probability**	**High probability**	***p*****-value**
Drug exposure	**Pre-Dose VDZ Concentrations (ug/mL) by Probability of Response**					
	Week 2	UC	22.9	27.4	32	<0.001
		CD	24.7	28.45	32.7	<0.001
	Week 6	UC	17.2	23.5	34.9	<0.001
		CD	15.3	23.5	33.4	<0.001
	Week 22	UC	18.0	23.8	32.5	<0.001
		CD	15.8	23.4	30.3	<0.001
	Week 46	UC	22.5	27.8	31.5	0.016
		CD	18.7	25.8	32.6	0.0008
Onset of action	**Change in Partial Mayo Score (UC) or Harvey-Bradshaw Index (CD) from Baseline by Probability of Response**					
	Week 6	UC	−1.22	−1.89	−2.21	<0.001
		CD	−1.69	−2.61	−4.22	<0.001
	Week 22	UC	−2.68	−3.2	−3.75	0.003
		CD	−3.76	−4.53	−5.82	<0.001
	Week 38	UC	−3.24	−4.21	−4.13	0.002
		CD	−4.62	−5.57	−6.76	<0.001
	Week 52	UC	−3.64	−4.42	−4.33	0.029
		CD	−4.68	−6.32	−7.17	<0.001

*AUROC, Area Under Receiver Operator Curse; CSFR, Corticosteroid-Free Remission; CSFER, Corticosteroid-Free Endoscopic Remission; CREM, Clinical Remission; CSF-REM, Corticosteroid-free Remission; MH, Mucosal Healing; DR, Deep Remission*.

*Dulai et al. CDST for CD Probability of response: Low (Intermediate ≤ 13), Intermediate (>13 to ≤19 points), High (>19 points)*.

*Dulai et al. CDST for UC Probability of response: Low (≤ 26 points), Intermediate (>26 to ≤32 points), High (>32 points)*.

## Future

Novel comparative head-to-head trials are forthcoming with the first such trial recently published. The VARSITY trial directly compared Vedolizumab vs. Adalimumab (ADA) as maintenance therapy in UC (33). Clinical remission rates at 52 weeks were 31.3% vs. 22.5% in VDZ vs. ADA (95% CI, 2.5–15.0; *p* = 0.006) and 52 week endoscopic improvement rates of 39.7% vs. 27.7% (95% CI, 5.3–18.5; *p* < 0.001). Rates of serious infections were low and similar between cohorts. This trial shows that VDZ is superior to ADA in achieving clinical remission and endoscopic improvement at 52 weeks maintenance therapy. Similar trials are sure to follow which will further inform on biologic positioning while adding more data to interpret predictors of response.

## Conclusion

VDZ is known to be safe, well tolerated, and effective. These are important points for personalization, but can we predict response to further guide therapy and shared decision-making? Subgroup analyses from the GEMINI trials were not powered for this question but they do provide evidence supplemented by real-world observational studies that increase generalizability for a heterogenous IBD population. Overall, it appears that patients with less severe disease (clinical, biomarkers) without prior biologic exposure and who demonstrate early response to VDZ have the highest rates of durable clinical and endoscopic response and remission. Prediction models and CDST confirmed these predictors and can be utilized to identify patients with higher probability of nonresponse so that either before initiation or after a short duration of treatment a decision to continue, discontinue, or even dose-escalation would be more informed. As biologics have become a mainstay of therapy, cost-analysis will help determine if prediction modeling can improve cost-effectiveness of VDZ by determining responders, nonresponders, and those with response latency needing dose escalation.

## Author Contributions

All authors listed have made a substantial, direct and intellectual contribution to the work, and approved it for publication.

### Conflict of Interest

PD is supported by an American Gastroenterology Association Research Scholar Award. PD has served as a consultant for Abbvie, Takeda, Pfizer, Janssen and received research support from Abbvie, Takeda, Pfizer, Janssen. The remaining author declares that the research was conducted in the absence of any commercial or financial relationships that could be construed as a potential conflict of interest.
